# Exceptional lymph node recurrence of an unusual ovarian tumor 16 years later: a case report

**DOI:** 10.1186/s13256-024-04476-5

**Published:** 2024-04-24

**Authors:** Saida Sakhri, Maher Slimane, Hanen Bouaziz, Nayssem Khessairi, Nadia Boujelbene, Tarek Ben Dhiab

**Affiliations:** 1 Department of Surgical Oncology, Salah Azaiez Institute, Faculty of Medicine of Tunis, University of Tunis El Manar, Boulevard 9 Avril 1938, Tunis, Tunisia; 2 Department of Pathology, Salah Azaïz Institute, Faculty of Medicine of Tunis, University Tunis El Manar, Tunis, Tunisia

**Keywords:** Sex cord tumor with annular tubules (SCTAT), Recurrence, Malignant, Therapy

## Abstract

**Background:**

Sex cord-stromal tumors with annular tubules are a rare tumor accounting for less than 1% of all ovarian malignancies. However, they are characterized by very late recurrence, which can be as late as 30 years after diagnosis and treatment.

**Case presentation:**

A 16-year-old female Caucasian patient was treated in our department for a stage IA ovarian sex cord-stromal tumors with annular tubules. She underwent a left salpingo-oophorectomy and ipsilateral pelvic node biopsy with no adjuvant treatment. She was seen for amenorrhea after being lost to follow up for 16 years. The diagnosis of recurrence was made by radiology and the elevation of serum inhibin B level. The patient underwent resection of the tumor, left segmental colectomy, and paraaortic lymphadenectomy because the mass was massively adherent to the left mesocolon. Histology confirmed the diagnosis with no metastatic lymph nodes. No adjuvant therapy was indicated. The patient was lost to follow-up again for 4 years and re-presented for amenorrhea. Serum inhibin B level was high. A second recurrence was suggested, and the patient underwent a laparoscopic surgery. We performed left pelvic and paraaortic lymphadenectomy, and 3 months after surgery the patient was pregnant.

**Conclusion:**

Sex cord-stromal tumors with annular tubules is a slow-growing ovarian tumor with a high potential for recurrence and metastasis. Surgery is the mainstay of treatment. Due to the rarity of these tumors, they are often unsuspected and thus incompletely staged before primary surgery; the diagnosis is made by histological examination. The prognosis of these patients is unknown, and they require long-term follow-up.

## Introduction

Sex cord-stromal tumors with annular tubules (SCTAT) are rare ovarian tumors accounting for less than 1% of all ovarian malignancies. They were first described by Scully in 1970 [1]. Until 1982, only 74 cases of SCTAT were reported in the literature [2, 3], and it usually occurs in the first two to three decades of life [4]. These tumors originate from stromal cells or primitive cells of the sexual cord and present a complex histology, making them a heterogeneous group of benign and malignant neoplasms. They are distinguished by the coexistence of simple and complex annular tubules, and have intermediate morphologic features between granulosa cell tumors and Sertoli cell tumors, so it can have focal differentiation into one of these tumor subtypes [5]. Two clinical subtypes have been defined: the first is associated with Peutz–Jeghers syndrome; this type is mostly benign, and it can be bilateral or multifocal, with the size usually being very small. The second subtype is sporadic; it may show malignant behavior and is usually unilateral and large in size [4].

Due to the rarity of these tumors, they are often unsuspected and thus incompletely staged before primary surgery, with the diagnosis being confirmed by histological examination [6]. The diagnosis is often made at an early stage and tends to be low-grade disease[7]. However, they have a high potential for late recurrence, which can be up to 30 years after diagnosis and treatment [4]. This case has been reported in line with the SCARE guidelines [8].

## Case presentation

We report the case of a 16-year-old female Caucasian patient, single, G0P0, who had menarche at the age of 13 years. No personal or family medical history to report. The patient consulted for amenorrhea preceded by an irregular cycle for 3 months. No melanocytic macules were seen on her lips. Ultrasonography revealed a left ovarian mass. Prolactin, thyroid function, estrogen, and androgen levels were normal. Serum levels of CA125 tumor antigen, α-foetoprotein, and sex hormone-biding globulin were also normal. She had undergone exploratory laparotomy, during which we discovered a left adnexal mass. No ascites or lymph nodes were observed. Contralateral ovary was normal, and no contralateral adnexal biopsy was performed. The patient underwent a left salpingo-oophorectomy without rupture of the mass. Histologic examination showed circumscribed columnar epithelial nests composed of ring-shaped tubules, which are encircled by hyalinized basement membrane-like material. No mitotic count was reported. The diagnosis of ovarian sex cord tumor with annular tubules staged IA Fédération Internationale de Gynécologie et d'Obstétrique (FIGO) was confirmed. No adjuvant treatment was administered. The patient was subsequently lost to follow-up.

**Fig. 1:**
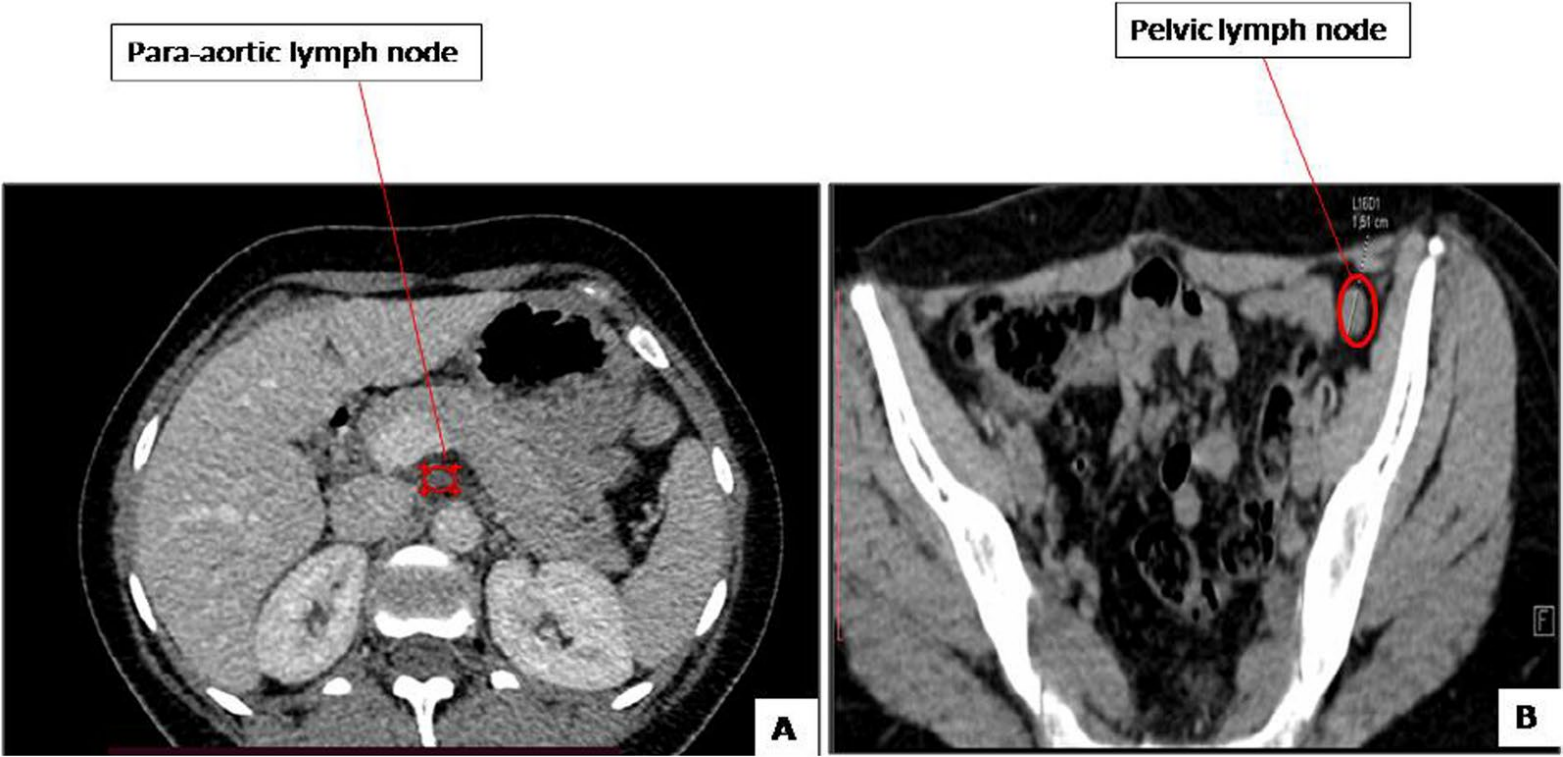
**A** Computed tomography scan showing paraaortic lymph nod (appointed with red arrow) e measuring 17 × 12 mm and **B** left pelvic lymph node (appointed with red arrow) measuring 16 × 5 × 10 mm

**Fig. 2:**
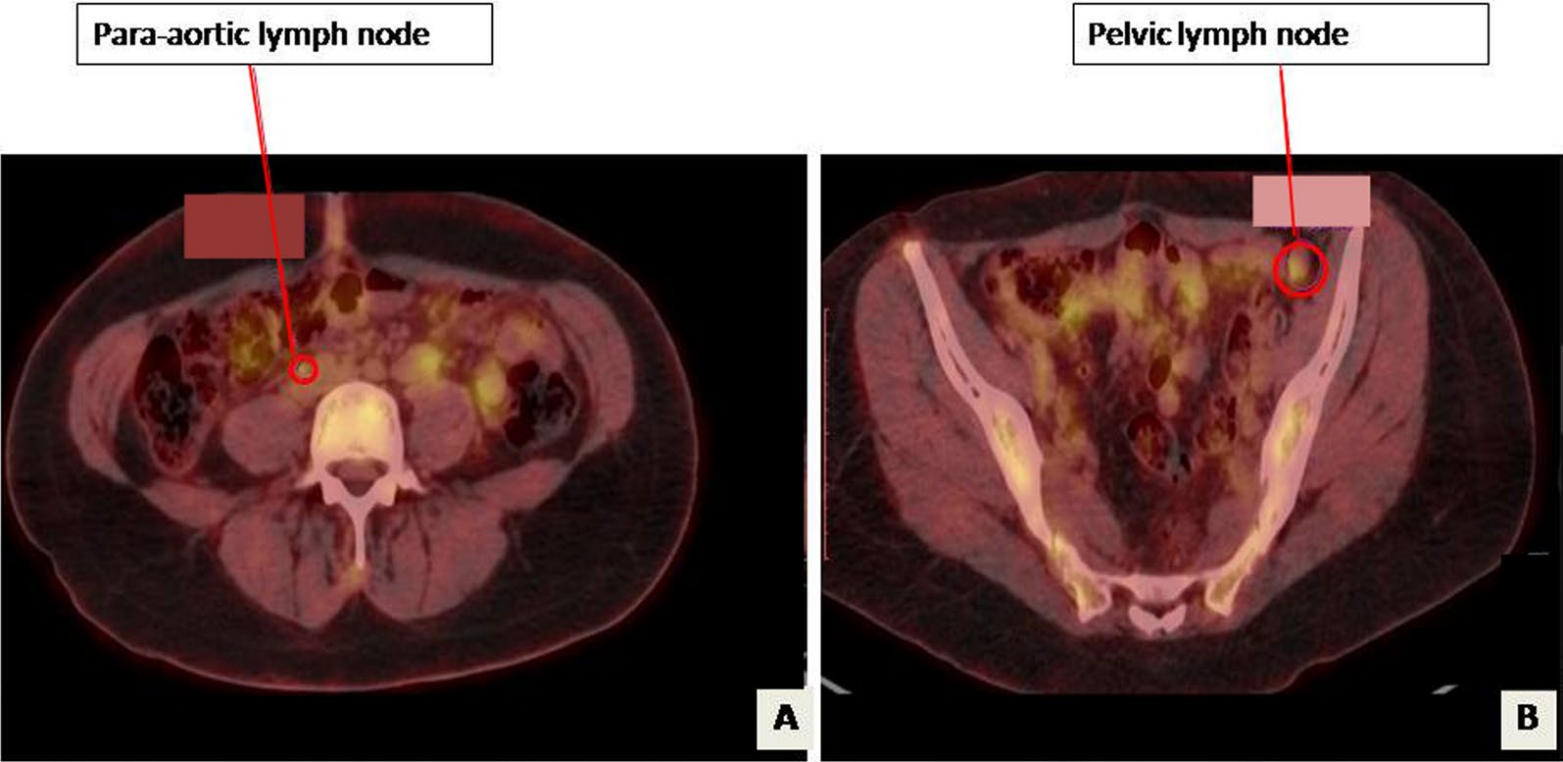
Positron emission tomography-computed tomography findings; **A** images show intense fluorodeoxyglucose uptake in paraaortic region (appointed with red arrow) and **B** in the pelvis lying (appointed with red arrow) along external iliac arteries

**Fig. 3:**
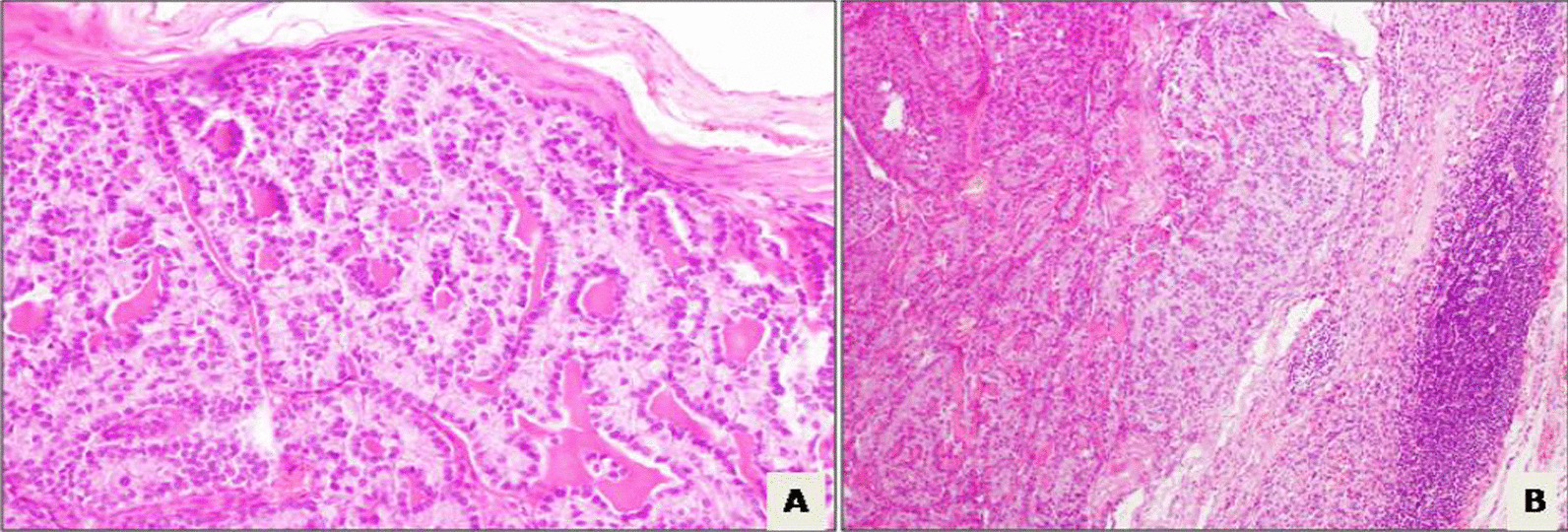
**A** Sex cord-stromal tumors with annular tubules characterized by annular tubular complexes filled with dense hyaline material. Tumor cells presented eosinophilic to pale cytoplasm with a round nuclei (Hematoxylin and eosin, 200 × magnification). **B** Massively invaded ganglionary parenchyma by sex cord-stromal tumors with annular tubules (Hematoxylin and eosin, 100 × magnification)

The patient presented 16 years later with amenorrhea. During this period, she was married at the age of 28 years and was pregnant at the age of 29 years, no details of the course and evolution of the pregnancy were available. Physical examination showed a left abdominopelvic mass of 20 cm without palpable adenopathy. Thoracoabdominopelvic computed tomography (CT) revealed a solid heterogeneous well-limited mass with calcifications situated in the left retroperitoneal region, measuring 16 × 14 × 10 cm. The mass was pushing back the left kidney and compressing the left ureter, leading to left hydronephrosis. Laboratory testing showed a high anti-Müllerian hormone (AMH) level at 5817 ng/ml. Serum inhibin B level was over 5000 pg/ml. Serum levels of CA125 tumor antigen, α-foetoprotein, and sex hormone-biding globulin were normal. A CT guided biopsy was performed. Histology examination confirmed the diagnosis of SCTAT recurrence. The patient underwent surgery by laparotomy. During intraoperative exploration, we found a voluminous retroperitoneal left encapsulated mass, with smooth limits, measuring 20 × 15 cm. The tumor was placed over aorta and intimately attached to the left renal vein and tail of pancreas without invasion. It was massively adherent to the left mesocolon. The right ovary and uterus were normal. We performed a resection of the tumor, of the left segmental colon, and paraaortic lymphadenectomy since there were palpable nodes in this region. There was no macroscopic residual tumor at the end of the operation. Final histological examination of the resection specimen confirmed the diagnosis with no metastatic lymph node. The postoperative course was free from complication. AMH and inhibin B levels decreased postoperatively at 2.44 ng/ml and < 8 ng/ml, respectively. The multidisciplinary medical committee decided that no adjuvant therapy was indicated. The patient was lost to follow-up again for 4 years. She then presented at the age of 36 years to our surgical department because of amenorrhea. Physical examination did not show any abnormalities: abdominal and gynecological examinations were normal. Laboratory testing showed a high serum inhibin B level at 717 pg/ml. Serum levels of CA125 tumor antigen, α-foetoprotein, and sex hormone-biding globulin were normal. AMH, estradiol, and progesterone testing were not performed. Abdominopelvic CT scan showed paraaortic lymph node measuring 17 × 12 mm and left pelvic lymph node measuring 16 × 5 × 10 mm; the uterine and the right ovary were normal (Fig. 1). We performed positron emission tomography (PET)-CT, which showed recurrence in pelvic lymph node (SUV = 4.2) and paraaortic lymph node (SUV = 3.4) (Fig. 2). The patient underwent laparoscopic surgery. During the intraoperative exploration, the left ovary, Fallopian tubes, and uterus, as well as the abdominal cavity, were free of nodes. The dissection of the left pelvic region uncovered the suspected lymph node, so we performed a left pelvic lymphadenectomy. In addition, the dissection of the paraaortic region uncovered the suspected lymph node, so we performed a paraaortic lymphadenectomy. The postoperative course was free from complications and the patient was discharged 3 days after surgery. Histological examination revealed one metastatic pelvic lymph node and one metastatic paraaortic lymph node for SCTAT without extranodal extension. The tumor was composed of a proliferation with annular, alveolar trabecular structures. Tumor cells presented eosinophilic to pale cytoplasm with round nuclei. The cells were arranged into peripheral placement. Hyaline and calcifications deposits were present as well as disseminated necrosis sites. This histological finding confirms the diagnosis of SCTAT (Fig. 3).

A multidisciplinary medical committee did not indicate adjuvant chemotherapy because the resection was qualified R0, given that the evolution of SCTAT is usually slow, and that the patient intended to conceive. The patient was regular to follow-up, and 3 months after surgery the patient was pregnant and lost to follow-up again.

## Discussion

Sex cord-stromal tumors with annular tubules (SCTAT) are mostly sporadic in two-thirds of cases, and they often occur at an early age, with average age of diagnosis of 36 years [1]. The diagnosis remains a clinical challenge due to the absence of specific symptoms. SCTAT are estrogen–progesterone secreting, and they show in 50% of cases symptoms of hyperesternism, irregular menstrual bleeding, postmenopausal bleeding, premature ovarian failure, fibroadenoma, and endometriosis. Therefore, serum progesterone and estradiol levels testing can be used for the diagnosis and the detection of recurrence. In fact, estradiol and progesterone concentrations drop dramatically after surgery, demonstrating the correlation between the upturn in concentrations and the onset of the disease [2, 9]. The main treatment is surgery, and fertility-sparing surgery is safety recommended at reproductive age. In our case, the main symptom was amenorrhea at the initial diagnosis and at the two recurrences, and we opted for the conservative surgery because of the young age of our patient. The rate of metastases in patients with sporadic SCTAT is around 20%, and they occur mainly in women of childbearing age [8]. However, the rate of recurrence ranges from 1% to 2.3% in the series [3]. In the study of Shen *et al*., the relapse and metastasis rate at first surgery was 20% [9]. Recurrence is generally ipsilateral to primary tumor and tends to occur several months to years after resection of the primary tumor. The average time to first recurrence was 45.5 months, ranging from 3 months to 20 years [3]. In our case, recurrence was ipsilateral and occurred after 4 years and 16 years. This may be explained by the non-reception of adjuvant treatment.

In the literature, despite the administration of adjuvant chemotherapy, the rate of recurrence is variable. Indeed, Shen *et al*. reported three cases of recurrence, of which two patients received adjuvant chemotherapy; the time to recurrence was longer in the cases that received adjuvant treatment [9].

Recurrence mostly occurs in the retroperitoneum, particularly affecting pelvic and paraaortic lymph nodes and other sites in retroperitoneum. Some authors reported supraclavicular lymph node metastasis; however, no metastasis was found in the uterus and in the contralateral ovary during the long-term follow-up [5]. In the present case, the two recurrences were ipsilateral, with the second recurrence being in pelvic and paraaortic lymph nodes despite the lymphadenectomy performed in the first surgery.

Malignant SCTAT may spread via lymphatic route with typical locations to pelvic, paraaortic, and supraclavicular lymph nodes. However, other less common areas may be affected, including the retroperitoneum, parietal and visceral peritoneum, liver, kidneys, and lungs [1, 2].

Recurrence can be detected by elevation of inhibin and Müllerian inhibitory substance (MIS), without elevation of CA125 or CEA. Inhibin and MIS have thus proved to be effective markers, correlating perfectly with the state of the pathology and generally returning to normal range whenever the patient is in remission [1, 2]. This finding is in line with our case, in fact, AMH and inhibin B levels were elevated in recurrence and decreased after surgery; however, there was no elevation of CA125 and CEA rates.

Laparoscopy is recommended for first surgery, and for the relapse. It can be also useful after neoadjuvant chemotherapy to evaluate the response of chemotherapy, although this response can be evaluated by PET scan and magnetic resonance imaging (MRI) [8]. Due to the rarity of these tumors, there are currently no standards for treatment. As for epithelial ovarian cancer, surgery is the major treatment option. Surgical management depends on the age of the patient, degree of ovarian involvement, and the desire to preserve fertility or ovarian function. Surgery should include ovarian cystectomy versus salpingo-oopherectomy [5]. The conservation of the ovary may increase the rate of recurrence, so some authors suggested salpingo-oopherectomy. However, in their retrospective study, Yinol *et al*. demonstrated that there is no statistically significant difference in recurrence rates between ovarian cystectomy and salpingo-oophorectomy (22.7% and 27.5%, respectively) [10]. In the current case, the patient underwent left salpingo-oophorectomy because the ovary was invaded; fertility preservation was still possible since the contralateral ovary was not removed despite the frequent recurrence.

Biopsy of the contralateral ovary is not routine, but it should be considered in case of suspicious lesion, unlike those are associated with Peutz–Jeghers [5]. Fertility-sparing surgery can be still indicated even in recurrent disease for patients with no involvement of contralateral ovary and uterus because, as shown in literature, it mostly occurs in adolescents or in women of reproductive age [2, 11].

The management of lymph nodes is unclear. Qian *et al*. suggested ipsilateral pelvic and paraaortic lymphadenectomy to reduce the rate of recurrence because most recurrences occur in the retroperitoneum [5]. Adjuvant chemotherapy is recommended in stages II–IV, and is usually based on cisplatin, etoposide, and bleomycin, thus demonstrating its benefit; in fact, it offered a good result in pathological complete remission [9].

Unlike other histological types of ovarian tumors, the first treatment of recurrence is surgery, consisting of resection of recurrent tumor with an aim to obtain an R0 resection. In some cases, such as extensive lesion and supra-diaphragmatic recurrence, chemotherapy is used firstly to reduce the size of masses so we can obtain complete resection. Even the protocol and the type of chemotherapy used remain controversial. Referring to the series, it was found that bleomycin, etoposide, and platinum (BEP) is usually used, as well as docetaxel, paclitaxel/ifosfamide, and paclitaxel/carboplatin, and rarely BEP combined with bevacizumab [2]. As the tumor has been shown to be hormone dependent according to some authors, recommended hormone therapy includes aromatase inhibitors, leuprolide, and tamoxifen. In case of complete or partial response, cytoreductive surgery may be considered [9]. The efficiency of adjuvant radiotherapy has not been proved, in fact, there are no randomized trials studying the effect of radiotherapy. Some authors use radiotherapy in case of supra-diaphragmatic recurrence, in addition to adjuvant therapy after local resection and for metastasis [9]. However, there are no details concerning the dose and effectiveness [1, 2]. Even with recurrence and metastasis, a diagnosis of SCTAT still has a good prognosis because most recurrences are well controlled with surgery and/or adjuvant treatment. The 5-year progression-free survival (PFS) is 92%. The median PFS time was 97.8 months. The 5-year overall survival (OS) was 100%. The risk of recurrence is still very high [5].

Prognosis depends on tumor clinical stage and tumor size. A tumor size of greater than 7 cm has been considered as the most important prognosis factor and mortality predictive factor. Additionally, the histological nature of neoplasm, whether benign or malignant, can affect prognosis [6, 9]. Due to the rarity of this entity, the mortality rate is not well established, but remains low, at around 1–2%[9]. Patients with SCTAT need to be followed for a long period, as recurrence occurs late in life. In case of pregnancy, the patient should be followed up regularly by a gynecologist and surgical oncologist, and monitoring includes inhibin levels and pelvic MRI to detect relapse. Unfortunately, our patient was lost to follow-up during her pregnancy.

## Conclusion

SCTAT is a slow-growing ovarian tumor that may exhibit malignant behavior with recurrent and metastatic potential, and recurrence occurs many years following initial diagnosis. The diagnosis should be made by expert gynecologic pathologists keeping in mind the wide differential diagnosis of other types of sex cord tumors. Surgery is the main treatment. The prognosis of these patients is unknown, and they should be followed up long term.

## Data Availability

Data supporting our findings were taken from the patient’s folders.

## Abbreviations

SCTAT: Sex cord-stromal tumors with annular tubules
AMH: Anti-Müllerian hormone
CT: Computed tomography
BEP: Bleomycin, etoposide, and platinum
